# Role of Stress-Survival Pathways and Transcriptomic Alterations in Progression of Colorectal Cancer: A Health Disparities Perspective

**DOI:** 10.3390/ijerph18115525

**Published:** 2021-05-21

**Authors:** Urbashi Basnet, Abhijeet R. Patil, Aditi Kulkarni, Sourav Roy

**Affiliations:** 1 Department of Biological Sciences, University of Texas at El Paso, El Paso, TX 79968, USA; ubasnet@utep.edu (U.B.); avkulkarni@utep.edu (A.K.); 2 Computational Science Program, University of Texas at El Paso, El Paso, TX 79968, USA; arpatil@miners.utep.edu; 3 The Border Biomedical Research Center, University of Texas at El Paso, El Paso, TX 79968, USA

**Keywords:** oxidative stress, antioxidants, apoptosis, gene regulation, microRNAs, biomarkers

## Abstract

Every year, more than a million individuals are diagnosed with colorectal cancer (CRC) across the world. Certain lifestyle and genetic factors are known to drive the high incidence and mortality rates in some groups of individuals. The presence of enormous amounts of reactive oxygen species is implicated for the on-set and carcinogenesis, and oxidant scavengers are thought to be important in CRC therapy. In this review, we focus on the ethnicity-based CRC disparities in the U.S., the negative effects of oxidative stress and apoptosis, and gene regulation in CRC carcinogenesis. We also highlight the use of antioxidants for CRC treatment, along with screening for certain regulatory genetic elements and oxidative stress indicators as potential biomarkers to determine the CRC risk and progression.

## 1. Introduction

According to the World Health Organization, there were approximately 1.8 million new colorectal cancer (CRC) cases and around 861,000 deaths recorded in 2018 across the world [1]. In the U.S., CRC is ranked as the second deadliest cancer [2]. A decline in both the incidence and death rate has been observed in the U.S. over the past few years [2]. The current improvements are believed to be related to an increase in the CRC screening rate and lifestyle modifications with less exposure to risk factors [3]. Though there has been a significant decline in the CRC incidence rate among older adults, there is an ongoing opposite trend among younger adults. It is estimated that the incidence of colon and rectal cancer among the younger adults within the age group 20 to 34 years could rise by 90% and 124.2% respectively, in the U.S., by 2030 [4]. The increased incidence rate in the younger population can be related to hereditary colorectal cancer syndrome, age, stress, high-fat and low-fiber diet, inactive lifestyle, tobacco smoking, or metabolic diseases [5,6]. However, precise etiologies behind the CRC incidence spike are yet to be discovered [4]. Along with the younger population, certain ethnic and racial minorities are also known to be disparities in CRC incidence and mortality as compared to Non-Hispanic Whites [7,8]. The susceptibility of these minority groups to CRC may be attributed to their lack of knowledge, inadequate healthcare facilities, or hereditary factors [9].

The malignant transformation of normal colonic mucosal cells is a multi-step process and may take 5 to 10 years [10]. Chromosomal instability and mutation in several tumor suppressor genes may lead to non-cancerous adenomatous polyps becoming cancerous in a majority of colorectal cancer cases [11]. Continuous genetic and epigenetic alterations, like DNA methylation or histone modification, in the normal colonic epithelial cells may lead to the development of colonic adenoma or adenocarcinoma [12]. In some cases, epigenetic regulators like microRNAs (miRNAs) are also involved in colon carcinogenesis, where they either inhibit the expression of tumor suppressor genes or promote the expression of oncogenes [13,14]. Interestingly, miRNA binding site polymorphism in inflammatory genes has been known to modulate the risk of CRC progression in different populations [15]. Genome-wide association studies have verified up to 52 independent loci that could regulate CRC development [16,17]. Additionally, certain genes regulating different pathways have been identified to contribute to the progression of CRC [18,19].

Besides genetic factors, extensive research is being conducted to determine the contribution of oxidative stress to the development of CRC [20]. The overaccumulation of reactive oxygen species (ROS) or reactive nitrogen species (RNS) in a cellular environment can damage the structure and function of the cells, leading to somatic mutation and neoplastic transformation [21]. Both ROS and RNS are highly reactive and are formed in vivo via oxidation-reduction reactions. These are essential in normal cells for signal transduction and phagocytosis [22]. However, ROS levels are usually higher in cancer cells compared to normal cells; and the imbalance in ROS production and the antioxidant defense mechanism typically results in irreversible cellular damage due to the oxidation of cellular macromolecules [23,24,25]. When present in enormous amounts though, they may initiate apoptosis or cell death leading to anticancer activity [26]. Altogether, it is evident that CRC carcinogenesis is a complex process, in which cross-talks between environmental and lifestyle factors lead to the regulation of multiple molecular pathways for its occurrence. These intricate interactions may also be the cause of resistance to drug or radiation-based therapies, implying a dire need for improved therapies to control one of the leading causes of cancer-based mortalities. In this review, we highlight the current health disparities in CRC and how gene regulation, oxidative stress pathways, and apoptosis relate to the progression of CRC. Further, we also discuss how these pathways and regulatory genes, including some miRNAs, could be used as biomarkers or therapeutic targets for CRC.

## 2. CRC and Health Disparity

According to the American Cancer Society, racial and ethnic minority groups in the U.S. have higher CRC-associated mortality in comparison to the overall population [7]. The colorectal cancer disparity is mostly based on socioeconomic status and race [8]. Though the incidence and mortality rate of CRC decreased annually by 3% from 2001 to 2010 nationally [3], the decline in CRC cases is not equally applicable to the entire population. The decline in CRC mortality is significant among Non-Hispanic White (males and females) compared to all other races [27]. There is a high incidence and mortality rate among African Americans when compared to Non-Hispanic Whites and Asians [7]. CRC is ranked as the second most common cause of mortality in the Hispanic population among all cancers [28]. Hispanics contribute to more than 17% of the total US population [29], but fall far behind when it comes to healthcare, as compared to the Non-Hispanic White population. As a result, Hispanic populations are usually diagnosed with colorectal cancer at a later stage, which limits their survival rate [30]. Since the progression of precancerous polyps to later-stage CRC is a prolonged process, early detection or screening for CRC could prevent the severity of the disease [31]. However, there are multifactorial reasons for CRC screening disparity among different populations in the U.S. [32]. Though African American men have the highest mortality rate due to CRC, the screening rate is much lower among this population. In the Hispanic population as well, CRC screening is conducted at a much lower rate compared to the Non-Hispanic White population. A study conducted in the Hispanic population living in El Paso, Texas, concluded that there is a lack of awareness and presence of perceived barriers for CRC screening in this ethnic group [33]. Other factors such as poor access to healthcare facilities, genetic predisposition to CRC, and the presence of higher CRC risk factors in the minority groups may also contribute to the higher statistics [9].

Globally, CRC is the third most diagnosed cancer. There is a higher risk of CRC in developed countries, but the incidence of CRC varies from region to region [34]. GLOBCAN studies have shown that there is a CRC gender disparity with a 50% higher cumulative risk in men as compared to women. The lesser incidence of CRC in females has been attributed to the female sex hormone, estrogen, which has adverse effects on the progression of CRC [35]. Though men are more susceptible to CRC, right-sided colon cancer (RCC) is more prevalent among females due to their ability to synthesize the amino acid asparagine abundantly and higher amino acid uptake [36]. Environmental factors, genetic and epigenetic changes, and distinguishing molecular features of males and females could also be the reasons for sex disparity in CRC [37]. *BRAF* mutation, one of the important factors in CRC is more commonly seen in females than males [38]. Besides demographic and racial backgrounds, 12% of colorectal cancer cases have also been related to consumption of the western diet [39]. Obesity among African Americans and Hispanics compared to Whites is considered as a predisposing risk factor for early onset of CRC [40]. A diet comprising highly processed food with high levels of fat, sodium, sugar, and low essential minerals and vitamins, is considered as a factor for the increasing rate of CRC cases [41]. Low CRC screening rates in Hispanic females who regularly consume alcohol [42], and the ability to metabolize alcohol due to genetic polymorphisms in enzymes involved in alcohol metabolism in Asians [43,44,45], have been correlated to a higher CRC incidence rate in these populations. Ethanol and/or its metabolites produced upon alcohol consumption are known to induce colon carcinogenesis by affecting lipid metabolism, epithelial to mesenchymal transition (EMT), angiogenesis, or adverse immune responses [46]. Expression of miRNAs such as *miR-34a*, *miR-21*, and *miR-135* is also seen to be altered by ethanol, affecting several oncogenic cell-signaling pathways linked to colorectal carcinogenesis [46]. 

Several methods have been employed to increase the screening rates in high-risk ethnic groups, including prioritizing physician recommendations, improved healthcare coverage, and patient navigators, but they have been met with heterogenous success [47,48]. Despite these measures, colonoscopy rates have increased only among Non-Hispanic Whites, and decreased among minority groups [49,50]. Previous studies have suggested that there is a higher rate of screening if the minority groups were offered the fecal occult blood (FOB) tests compared to colonoscopy [51]. However, there is a geographic variation observed in the availability and acceptance of the FOB testing method. Further studies are required to decipher the receptiveness of the FOB test in minority groups across the U.S., which would provide a better comparison with colonoscopy and aid in improving their participation in routine CRC screenings.

## 3. Genetic Alterations in CRC

CRC is a multistep process that is described by a shift in the gene expression profile when the cell starts to evolve from the early to the late stage of CRC [52]. In this section, we focus on some of the genes that have been identified to be related to tumorigenesis and metastasis of CRC (Table 1). The cell cycle and p53 signaling pathways are seen to be among the top enriched pathways in CRC transcriptomes [19]. The genes involved in the cell cycle promote the endothelial cell proliferation that contributes to the tumor progression and metastasis of CRC [53]. One of the key regulators, cyclin-dependent kinase 1 (*CDK1*), is seen to be highly enriched in CRC patients [54]. Another cell cycle gene, Cyclin A2 (*CCNA2*) shows higher expression in CRC than in normal tissues, and knockdown of *CCNA2* is known to suppress the CRC cell growth significantly by impairing the cell cycle progression and promoting cell apoptosis [55]. Alterations in the p53 signaling pathway due to mutations in the *TP53* gene are also associated with the loss of the transactivation feature in advanced CRC cases, which leads to poor survival [56]. However, activation of p53 may also be related to cellular stress, which could target downstream genes that can influence apoptosis, cell cycle arrest, and angiogenesis through mRNA:miRNA interactions [57]. The ribonucleotide reductase regulatory subunit m2 (*RRM2*) gene, which is a part of the cell cycle and p53 signaling pathway, is also known to be overexpressed in CRC [18], and has been correlated with the poorly differentiated type, invasion depth, and tumor node metastasis stages in CRC [18]. Among the other cell cycle and p53 signaling pathway genes are DNA topoisomerase 2-alpha (*TOP2A*) [58], cell division cycle 6 (*CDC6*) [59,60], nucleolar and spindle associated protein 1 (*NUSAP1*) [61], centrosomal protein 55 (*CEP55*) [62], budding uninhibited by benzimidazoles 1 (*BUB1*) [63], and the mitotic arrest deficient 2 like 1 (*MAD2L1*) [63], which are known to be differentially expressed in CRC, and are related to the aggressive tumor phenotypes and advanced tumor stages.

The risk of CRC carcinogenesis has also been associated with genes modulating the oxidative stress pathway. The genes encoding for peroxiredoxin 1 (*PRDX1*), glutathione peroxidase 1 (*GPX1*), and superoxide dismutase (*SOD*) have been seen to be highly upregulated in CRC samples [92,105,106,107,116]. The proteins encoded by these genes are known to be crucial for oxidative balance in cells; however, their role or mechanism of interaction in CRC metastasis and angiogenesis remains understudied [105,116]. For decades, the use of manganese-SOD or zinc/copper-SOD is under evaluation as treatment(s) for CRC. However, there has been limited evidence about their prognostic value from either of the clinicopathological stages of CRC [92]. Recent studies suggest, PRDX1 reduces the production of pro-inflammatory cytokines IL-8 and CXCL1 [106], or interacts with poly(ADP-ribose) polymerase (PARP) tankyrase to promote tumor development via the adenomatous polyposis coli (*APC*) gene [107]. However, further studies to confirm PRDX1 as an inflammation marker in CRC development or as a therapeutic target against inflammation-associated CRC need to be conducted.

Apart from the genes associated with stress survival pathways, genes encoding for different kinases have also been seen to be differentially expressed in various CRC cases. The top candidates include serine/threonine-protein kinase (*PLK1*) [19,103], aurora kinase A (*AURKA*) [63], checkpoint kinase 1 (*CHEK1*) [78], and maternal embryonic leucine zipper kinase (*MELK*) [100]. These genes are seen to control the proliferation, migration, invasion of CRC cells, prognosis, or metastasis of CRC. Furthermore, genes encoding for cytokines belonging to the CXC family are crucial in regulating inflammation and angiogenesis in CRC [117]. Studies have identified *CXCL1*, *CXCL3*, and *CXCL8* as the hub genes in the development of CRC [118]. Moreover, peptide tyrosine-tyrosine (*PYY*), calcium-activated chloride channel 4 (*CLCA4*), hematopoietic cell-specific lyn substrate 1 (*HCLS1*), ecotropic viral integration site 2B (*EVI2B*), and cluster of differentiation 48 (*CD48*) are also some of the genes differentially expressed in CRC tissues [90,118]. Apart from differential gene expression patterns, a few genetic mutations have also been correlated with CRC incidence in populations of different ethnicities. Mutations in two genes, ephrin type A receptor 6 (*EPHA6*) and folliculin (*FLCN*), have been identified as CRC drivers in the African American population [119].

In the current era with advances in high-throughput genomic technologies, extracting information about specific gene expression profiles has become highly attainable. However, not many studies have been conducted to use this information to screen for potential therapeutic targets or biomarkers. Studying the biological significance of the expression of these genes in CRC progression and in promoting tumor aggressiveness or studying their influence on the tumor microenvironment would help design robust diagnostic tools or identify therapeutic targets for CRC in the future. For example, as *CDK1* is known to be enriched in many cancer types, including breast cancer [120], pancreatic ductal adenocarcinoma [121], oral squamous cell carcinoma [122], and hepatocellular carcinoma [123], consideration of *CDK1* as a potential biomarker may be advisable. Though oncogenic long non-coding RNA *MALAT1* is considered as a poor prognostic indicator [124], the expression of *MALAT1* is seen to be significantly higher in African Americans compared to CRC tissues of Caucasians, making it a marker for disparate CRC incidence and severity in African American population [125]. Microsatellite instability (MSI) has been used as a biomarker for detecting defective DNA MMR in CRCs [126,127,128]. It is typically assessed by analyzing at least five microsatellite markers: three dinucleotides (D2S123, D5S346, D17S250) and two mononucleotides (BAT25 and BAT26) repeats, as suggested by the National Cancer Institute’s Bethesda panel [129]. Due to lower sensitivity rates, new quasi-monomorphic mononucleotide markers, known as the pentaplex panel have been applied [130]. Studies have shown differences in the MSI frequencies in African Americans and Puerto Rican Hispanics as compared to Caucasians [131,132]. However, no significant difference has been reported in a recent study comparing the frequency of MSI in different ethnic groups [133]; implying the need for evaluating the methodologies and biological sources used in other studies for further use of MSI as genetic markers. Investigating the role of oxidative stress-inducible genes as biomarkers or therapeutic targets would also provide insights into controlling CRC metastasis. From a therapeutic perspective, *HCLS1*, *EV12B*, and *CD48* genes are seen to be negatively associated with the prognosis of CRC patients [90]. Exploring the effects of overexpression of *HCLS1*, *EV12B*, and *CD48* genes in reducing migration, proliferation, and invasion of CRC cells and eventually suppressing CRC tumor growth could provide us with promising CRC therapies [90]. Further, deciphering ways to overcome TOP2A [58] or CXCL8 [134] mediated chemotherapeutic resistance is also of acute importance.

## 4. Role of microRNAs in CRC Carcinogenesis

Information-carrying biomolecules such as miRNAs, extracellular vesicles (or exosomes), circulating tumor cells, and cell-free DNA are known to regulate CRC metastasis through epithelial-mesenchymal transition, angiogenesis, immunosuppression, and chemoresistance [135,136,137,138,139,140,141,142]. Because of their importance in CRC carcinogenesis, these are considered potential biomarkers for disease progression and response to cancer therapies [135,136,137,138,139,140,141,142]. Though all of these have an excellent basis for use as genetic markers, due to their atypical expression patterns detected during CRC carcinogenesis, we focus on the roles of miRNAs in CRC progression and response to therapy in this section [135,136]. These are a family of small non-coding single-stranded regulatory RNAs comprising of 19 to 25 nucleotides. They regulate gene expression by either mRNA degradation or by suppressing translation [143,144]. Though their role in tumor regulation has been known for more than 20 years [145], they have only recently been considered as a tool for CRC screening due to their aberrant expression during colorectal carcinogenesis [135,136]. Many microarray studies have shown that the miRNA profiles from healthy controls, patients with colorectal adenoma, and CRC are significantly different [146,147]. Some of the miRNAs (Table 2), *miR-21*, *miR-92a*, *miR-135b*, *miR-18a*, *miR-18b*, *miR-31*, *miR-212*, *miR-431*, and *miR-503*, have been identified to be upregulated in different stages of CRC contributing to the transformation of normal mucosa to CRC [147,148,149]. On the contrary, *miR-14*, *miR-451*, *miR-638*, *miR-133a*, *miR-375*, *miR-378*, *miR-422*, and members of the *miRNA-320* family are seen to be significantly downregulated in colorectal adenoma and CRC tissues [147,149,150]. These miRNAs are thought to target important cell cycle genes such as *CDK6* [150], RAS p21 protein activator 1 (*RASA1*) [151], or *KRAS* gene [152] to promote cell proliferation, migration, and invasion in CRC. Approximately 230 miRNAs are differentially expressed in colorectal adenocarcinoma samples collected from patients that had a pre-existing adenoma [153]. When compared with survival rate, nine key miRNAs, *miR-125a, miR-125b, miR-328, miR-129, miR-217, miR-375, miR-486, miR-194,* and *miR-144*, have been seen to be differentially expressed and had a direct correlation with survival rates in different colorectal adenocarcinoma cases [154]. In a small-scale study among the thirty African Americans and thirty-one Caucasian CRC subjects, five miRNAs (*miR-182, miR-152, miR-204, miR-222,* and *miR-202*) were found to be correlated to racial tumorigenesis [155]. Among these five miRNAs, *miR-182* was observed to be highly upregulated in CRC tumors of African Americans compared to Caucasians; contributing significantly to the roles of miRNA in CRC racial disparity [155]. While the observations from this study regarding increased *miR-182* expression in African Americans need further confirmation in larger sample sets along with better annotation of racial metadata in existing collections, it does provide evidence for racial differentiation in miRNA expression. 

As many studies are identifying the roles of miRNAs in the occurrence and development of CRC, miRNA expression profiling may be a new alternative approach to monitor the transition of colorectal adenoma to carcinoma [170]. This may also help to overcome the issues with traditional ways of monitoring or diagnosing the progression of CRC through colonoscopy and/or fecal occult blood tests, as these methods are expensive, invasive, and not sensitive [170,171,172]. Recent studies have shown that expression levels of *miR-21, miR-106a, miR-7, miR-17, miR-21, miR-92a, miR-96, miR-134,* and *miR-196a* are significantly upregulated in the stool samples of CRC patients [173,174]. Intriguingly, the expression levels of some of these miRNAs were found to be even higher in patients with later tumor-lymph node metastatic (TNM) carcinoma stages compared to those with adenomas [174]. Furthermore, some miRNAs (*miR-9*, *miR-138*, *miR-143*, *miR-127-5p*, *miR-29b*, *miR-938*, and *miR-222*) were significantly downregulated in the stool samples of colorectal cancer patients [174]. Implementing screening of these miRNAs for clinical diagnostic purposes would provide a promising non-invasive approach to detect CRC in the future.

## 5. Role of Oxidative Stress and Antioxidants in CRC Progression

The link between oxidative stress and colorectal cancer has been studied vigorously over the past decade. Oxidative stress is an imbalance of oxidants or reactive oxygen species (ROS) and antioxidant defense in the cell. It can be produced both intracellularly or extracellularly [175,176]. Intracellularly, the production of ROS is dependent on almost all enzymes that use molecular oxygen as a substrate like NADPH oxidase, microsomal cytochrome P450, peroxisomes, xanthine oxidase, cytokines, and growth factors. Besides ROS, reactive nitrogen species (RNS) could also contribute to redox signaling in cells. Several external factors such as UV light, smoking, air pollution, stress, or medication could also enhance the free radical formation [177]. At lower concentrations, these free radicals are necessary for the host defense mechanism and tissue repair mechanism [178]. However, when present at higher concentrations, they may lead to mutations in the DNA or neoplastic transformation, causing cellular damage [179,180]. The evolution of cancer has been correlated with oxidative stress-induced DNA damage and genetic instability [21]. Superoxide anion, hydroxyl radical, hydroperoxyl radical, and hydrogen peroxide from different intracellular metabolic pathways majorly contribute to intracellular ROS levels [181], leading to lipid peroxidation, protein oxidation, or DNA damage; eventually progressing to the development of cancer [182,183,184]. In DNA, ROS can induce single or double-strand breaks, or nucleotide base modification [24]. The final products of lipid peroxidation such as malondialdehyde (MDA) and 4-hydroxy-2-nonenal (4-HNE), can also react with the DNA bases to give rise to DNA adducts, which could promote CRC carcinogenesis [20]. DNA repair proteins such as glycosylases, endo and exonucleases, DNA ligases, and DNA polymerases contribute to the removal of the oxidized base pairs by either base excision repair (BER) or MMR. The damage caused by 5-fluorouracil (5-FU), a common chemotherapeutic drug used for CRC treatment, is also repaired by BER or MMR [128,185,186], which represents the patients DNA repair capacity (DRC) determining their response to chemotherapy and CRC prognosis [128,185,186]. Studies have been conducted to determine the use of BER-DRC as a prognostic marker to 5-FU therapy [128]. However, evidence suggests that these DNA repair proteins may also be disrupted due to higher levels of ROS [187] or subjected to altered activity due to DNA polymorphisms [188].

Cells can react to oxidative stress in different ways; creating stress survival strategies if the exposure is limited. However, if the cell goes through prolonged perturbation against the natural strategies for this balance, the cells either become apoptotic or undergo necrotic cell death [189,190]. There is an involvement of several ROS metabolizing enzymes such as catalase, peroxiredoxins, glutathione peroxidase, superoxide dismutase, glutathione reductase, and thioredoxin reductase to maintain pro-oxidants: antioxidants balance [191]. As mentioned in the previous section, genes encoding for some of these proteins, GPX1, PRDX1, and SOD, are known to be upregulated in CRC samples [92,105,106,107,116]. Non-enzymatic antioxidants such as glutathione (GSH), coenzyme Q, uric acid, polyphenols, and melatonin may also work in a complex system with enzymatic antioxidants to reduce ROS levels [183]. Antioxidants such as gamma-tocopherol, carotenoids, tocotrienols, aspirin, and vitamin C have also been linked to a reduced risk of CRC [175,192,193]. For the beneficial effects of such antioxidants in preventing carcinogenesis, efforts are being made to identify their source and mode of action [175].

For years, it has been well established that oxidative stress is one of the major contributors to CRC progression [194]. However, previous work does not contribute to affirming the role of antioxidants in reducing oxidative stress in human subjects with a high risk of CRC [175]. It is evident that not all compounds with antioxidant capacity could reduce the risk of CRC progression; however, critical evaluation of optimal food antioxidants for preventing CRC is crucial. Only a few studies have been conducted to detect increased levels of oxidative stress markers and antioxidants in the blood of patients with CRC [194,195,196,197]. Several studies have been conducted to detect oxidized nucleotides in the urine sample of patients diagnosed with breast and lung cancer, atherosclerosis, and diabetes [198,199,200,201,202,203,204]. DNA oxidation, measured as urinary excretion of 7,8-dihydroxy-8-oxoguanosine (8oxoGuo) has been proven to be a prognostic tool for these diseases [201,202,203,204]. Further, detection of 8oxodG, has recently been emphasized as a marker for a high level of RNA oxidation in neurodegeneration, breast cancer, and diabetes [198,199,200]. Implying that the findings from these studies would be interesting for the development of new diagnostic tools for CRC development and progression. Studies in small populations have shown that the measurement of reactive oxygen metabolites or the ferric reducing ability of plasma as ROS production biomarkers could be promising diagnostic tools [194,195]. Malondialdehyde, 4-hydroxy-2-nonenal (4-HNE), and isoprostanes, which are the final products of polyunsaturated fatty acids are currently being used as biomarkers for CRC detection [196]. However, long-term follow-up remains a consistent issue in human subject trials [194]. It is essential to emphasize longer follow-up durations and repeated measurements of both ROS and antioxidant levels in larger populations to explore their use in clinical practice. Substantial work also needs to be done to understand the complex network governing DNA damage, and genotoxic effects of ROS in the progression of CRC.

## 6. Apoptosis and CRC Progression

Apoptosis is the process of programmed cell death or cell suicide that maintains the cell population in the tissue [205]. It helps to get rid of unwanted cells during developmental stages or the cells with damaged DNA or cytoskeleton which are beyond repair in adults [206]. If apoptosis is for some reason interrupted, inappropriate apoptosis can lead to uncontrolled cell division and the subsequent development of a tumor. Its ability to modulate the life or death of a cell is recognized for its immense potential to be used as a therapeutic target. In normal colon epithelial cells, to preserve the structure, a balance is maintained between apoptosis at the top of the crypts and cell proliferation at the base of crypts [207]. The increased apoptosis rate at the bottom of the crypts and higher proliferation rate at the top of crypts in adenomas hinder the colonic epithelium homeostasis that is correlated with the progression of CRC. However, in carcinomas, apoptosis is not specifically localized [208]. During the evolution of colon cancer from adenomas to carcinoma, differential expression of many apoptosis-related proteins is observed, indicating the importance of apoptosis in colorectal carcinogenesis [209]. Tumor suppressor genes (*APC* or *TP53*) and oncogenes (*KRAS* or *BRAF*) work together in apoptotic failure and progression of CRC [206,210,211]. These genes may sometimes also regulate sporadic and hereditary CRC [52]. Up to 70% of CRC cases show either mutation or deletion in tumor suppressor genes including *TP53* [209]. Activated p53 can upregulate the expression of genes that encode pro-apoptotic BCL-2 proteins such as Noxa, PUMA, Bax, and some death receptors such as Fas, PIDD, DR5 [211]. When a ligand binds to death receptors such as Fas and DR5, it initiates the activation of the caspases, which further turns on the apoptotic cell death pathway [212]. The *KRAS* gene is another commonly mutated gene present in approximately 40% of CRC cases [213]. Interestingly, the *KRAS* mutation is more frequently found in African Americans than Caucasian colonic tumors [214], but the rate of BRAF mutation is twice as much in whites compared to Asians or African Americans, with Asians having the least rate of occurrence [214,215]. Currently, the mediators BCL-2, DR-5, and caspases are being used as targets for testing the efficacy of drugs for CRC treatment in clinical and pre-clinical trials [216]. However, dysregulated apoptosis in the colonic epithelial cells also contributes to radiation and chemotherapy resistance [207]. Apoptosis is an important regulatory process and pro-apoptotic proteins such as the BCL-2 family of proteins may function as monitors of damage signals, it would be beneficial to further explore their potential as logical targets to overcoming drug resistance.

## 7. Conclusions

Colorectal cancer remains one of the leading causes of mortality due to cancer. Despite the decline in CRC cases in the U.S. over the last few decades, some ethnic groups still have a higher incidence and mortality rate. Knowledge of CRC health disparity among different ethnicities is crucial to identify and improve the potential risk factors for CRC. Very few studies have been conducted to elucidate the molecular basis of CRC carcinogenesis in different ethnic groups. One of the major contributors to CRC carcinogenesis is oxidative stress. Here, we have elucidated the importance of oxidative stress in CRC progression, and the function of antioxidants as a preventive measure. We have also focused on genes that may contribute to the development and progression of CRC, as they could be considered biomarkers and therapeutic targets. Since CRC is a multifactorial disease, a better understanding of molecular pathways involved in CRC initiation and progression is vital for the prognosis and treatment of CRC. Conducting further studies in minority groups to identify genetic markers for CRC predisposition is critical and would be an interesting area to explore in the future.

## Figures and Tables

**Table 1:** Differentially expressed genes during CRC carcinogenesis.

Gene	Function(s)	Role in CRC Progression	Pathway	UR/DR
*AURKA*	Regulates mitotic spindle formation [64].	Contributes to malignant transformation of colorectal adenomas to carcinoma [65,66].	Cell cycle	UR
*BUB1*	Assembles spindle checkpoint proteins at kinetochore, and is required for chromosome alignment [67].	Mutations in the *BUB1* gene cause chromosome missegregation during CRC progression [68]. However, the role of BUB1 in CRC development remains poorly understood.	Cell cycle	UR
*CCNA2*	Activates CDK1 and CDK2 to promote somatic cell division [69,70].	Promotes G1/S and G2/M phase transitions in CRC cells with reduced apoptotic cells [55].	Cell cycle	DR
*CD48*	Activate T cells, antigen-presenting cells and granulocytes [71].	Target of NF-κB signaling during CRC invasion [72].	Innate immunity	DR
*CDC6*	Required for formation of the pre-replicative complex. Regulates G1, S, and mitosis phases in the eukaryotic cell cycle [73].	Human antigen R (HuR) regulates CDC6 activity to promotes cell proliferation with increased DNA synthesis, epithelial-mesenchymal transition (EMT), migration, invasion in CRC, and confers resistance to oxaliplatin [60].	Cell cycle	UR
*CDK1*	Controls cell cycle by regulating mitotic onset [74].	Phosphorylates JAK1 and triggers JAK/STAT3 signaling pathway to promote CRC metastasis [75].	Cell cycle	UR
*CEP55*	Centromere protein that is required for mitotic exit and cytokinesis [76].	Activate p53/p21 axis to promote CRC proliferation and metastasis, and mutation in *CEP55* gene have been related to overall CRC patient survival [62].	Cell cycle	UR
*CHEK1*	Regulates checkpoint-mediated cell cycle arrest, and DNA repair in response to any DNA damage [77].	Reduced expression of *CHEK1* has been speculated to be an important inactivating mechanism for impaired DNA polymerase function and the development of colorectal neoplasm [78,79].	Cell cycle	DR
*CLCA4*	Calcium activated chlorine transport [80].	Inhibits cell invasion and migration through suppression of EMT via the PI3K/AKT pathway in CRC [80].	Ion channel transport	DR
*CXCL1*	Recruits neutrophils to activate the host immune system for microbial killing [81,82].	Proinflammatory mediators such as prostaglandin E2 are thought to induce CXCL1 targeted angiogenesis in CRC [83].	Phagocytosis and inflammation	UR
*CXCL3*	Chemotactic activity for neutrophils [84].	Gene set enrichment analysis in CRC samples has revelated that elevated *CXCL3* levels could be associated with impaired DNA repair, cell cycle process, cell apoptosis process, and the p53 regulation pathway; however, further studies are required to decipher the prospective molecular mechanism [85].	Phagocytosis and inflammation	UR
*CXCL8*	Acts as a chemotactic factor that attracts neutrophils, basophils, and T-cells, but not monocytes [86,87].	Induces EMT of CRC cells to aid in evasion host immunosurveillance and enhance anoikis resistance to promote distant organ colonization [88].	Phagocytosis and inflammation	UR
*EVI2B*	Control granulocyte differentiation and functionality [89].	CRC proliferation, migration, and invasion have been correlated with reduced expression of *EVI2B* [90]. Functional studies to determine downstream molecular target(s) need attention.	Cell cycle	DR
*GPX1*	Catalyzes the reduction of hydrogen peroxide to water and oxygen [91]	Though the levels of *GPX1* are seen to increase in CRC tissues [92], possible modes of action of these enzymes in CRC progression need further investigation.	Oxidative stress	UR
*HCLS1*	Plays an important role in lymphocyte trafficking, neutrophil chemotaxis, and growth arrest [93,94,95]	Target of *miR-1296-5p* mediated endonucleolytic cleavage in CRC carcinogenesis [96].	Cell cycle and immunity	DR
*MAD2L1*	Chromosome alignment at the metaphase plate [97].	The expression of *MAD2L1* is seen to increase gradually with the stages I-IV of CRC [98], suggesting it could be important for tumor progression, but its clinical function in CRC is still unknown.	Cell cycle	UR
*MELK*	Interacts and phosphorylates with BCL-G, CDC25B, MAPK, NIPP1, p53 to regulate the cell cycle, self-renewal of stem cells, and apoptosis [99].	Phosphorylates AKT through FAK/Src pathway to increase proliferation, migration, and invasion of CRC cells [100].	Cell cycle	UR
*NUSAP1*	Microtubule organization and chromosome segregation during cell division [101].	Promotes cell proliferation, migration, invasion, and EMT in CRC via DNA methyltransferase 1 (*DMT1*) expression [61].	Cell cycle	UR
*PLK1*	Performs several important functions during mitosis along with mitotic exit and cytokinesis [102].	Acts as an indispensable protein in cellular mitosis and proliferation, and is crucial for migration and invasion in CRC [103].	Cell cycle	UR
*PRDX1*	Protects the cells against oxidative stress by reducing peroxides to oxygen and water [104].	Upregulated *PRDX1*, increases the production of matrix proteins (MMP2/MMP9) or growth factors (VEGFA) [105], reduces the production of proinflammatory cytokines and chemokines (IL-8 and CXCL1) [106], or interacts with PARP tankyrase to promote tumor development via APC [107].	Oxidative stress	UR
*RRM2*	Synthesizes deoxynucleotides from ribonucleotides for DNA polymerization and repair, and supplies dNTPs for mitochondrial DNA replication and repair via p53 [108,109,110].	Regulates infiltration and metastasis by increasing hyperplasia and cell invasion [18].	Cell cycle, p53 signaling	UR
*SOD1*	Converts superoxide radicals into hydrogen peroxide and oxygen [111].	SOD production levels are seen to increase proportionally with CRC severity [112]; though, further studies are required to determine the downstream functional pathway.	Oxidative stress	UR
*TOP2A*	Regulates chromosome condensation, chromatid separation, transient breaking, and rejoining of DNA strands during transcription and DNA replication by altering the DNA topology [113].	High copy numbers have been associated with mismatch repair (MMR) competent CRC patients [114], but have also been associated with aggressive and advanced CRC phenotypes due to their ability to inhibit apoptosis and induce drug resistance [58].	Cell cycle	UR
*TP53*	Tumor suppressor protein that acts as a transcription factor to regulate cell division and DNA repair [115].	Mutations in the TP53 gene, impair the transactivational ability of p53 to initiate downstream gene expression to regulate cell cycle arrest, apoptosis, and angiogenesis [56,57].	p53 signaling	DR

DR—Downregulated; UR—Upregulated.

**Table 2:** Important miRNAs in CRC carcinogenesis.

miRNA	Role(s) in CRC Progression	Target(s)	UR/DR
*miR-18a*	It is the most conserved and multifunctional miRNAs and is often overexpressed in tumors [156]. *miR-18a* has a dual functional role in either promoting or inhibiting tumorigenesis in many human cancers including CRC [156].	*IRF2, PTEN* *SOX6, WNK2* *STK4, PIAS3* *CDC42*	UR
*miR-21*	Located within the intronic region of the *TMEM49* gene. It plays a crucial role in many biological functions and has been upregulated in many diseases including cancer, cardiovascular diseases, and inflammation [157]. *miR-21* also plays important roles in carcinoma-associated fibroblast formation, tumor formation, and metastasis [158].	*SMAD7* *PDCD4* *TPM1* *CDC25a* *TIMP3*	UR
*miR-31*	*miR-31* has been shown to increase cell growth in CRC cells and also stimulates oncogenesis by repressing *RASA1* [151].	*RASA1*	UR
*miR-92a*	Also known as oncomiR, it enhances cell proliferation, induces tumor angiogenesis, suppresses apoptosis of cancer cells, and promotes tumor progression in various cancers including CRC [15]. Upregulated expression of *miR-92a* in plasma or stool has shown to be effective in predicting CRC [159].	*PTEN* *SMAD2* *SMAD4* *TGFBR2*	UR
*miR-125a/b*	*miR-125* has shown to be involved in multiple cancers. It is downregulated and inhibits cell proliferation, migration, and invasion in CRC [160].	*P53, PUMA* *BAK*	DR
*miR-126*	It contributes to cell proliferation, invasion, and progression of angiogenesis. In highly metastatic CRC cell lines, the expression of *miR-126* is known to be significantly reduced [15,161,162].	*P13K, CXCR4, VEGFA, IRS1*	DR
*miR-135b*	It is common in sporadic and inflammatory bowel disease-associated human colorectal carcinomas, and is correlated with tumor stage [163]. *miR-135b* has a role in the early stages of CRC [136].	*APC*	UR
*miR-143*	*miR-143* regulates cell growth and proliferation [164]. It is a tumor suppressor miRNA. It suppresses cell growth and proliferation by repressing the translation of *KRAS* and *DNMT3A* [136].	*DNMT3A* *KRAS* *CD44*	DR
*miR-155*	It is overexpressed in CRC and lung cancer. Mediates cell proliferation, invasion, and angiogenesis [165]. It is also linked with drug resistance, poor prognosis, and genome instability in CRC patients [166]. *miR-155* suppresses the expression of *PTPRJ,* affecting cell proliferation and migration in CRC [15,167].	*PTPRJ* *TP53INP1* *MSH2, MSH6* *FOXO3*	UR
*miR-194*	It is closely associated with the overall survival of CRC patients, tumor size, and tumor node metastasis (TNM) [15]. It suppresses tumor growth by regulating the MAP4K4/c- Jun/MDM2 signaling pathway [168].	*MAP4K4, AKT2*	DR
*miR-499*	*miR-499* promotes cell migration and invasion in CRC cell lines by targeting *FOXO4* and *PDCD4* [169].	*FOXO4* *PDCD4*	UR

DR—Downregulated; UR—Upregulated.

## Data Availability

Not applicable.
